# Spasmodic Croup

**Published:** 1901-03

**Authors:** 


### Spasmodic Croup.

℞ Sodii carbonatis........................ ʒ ij.

Aquæ.......................................... ℥ iv.

M. S. Use for half an hour in a steam atomizer.

